# Comparison of different cytogenetic methods and tissue suitability for the study of chromosomes in *Cimex lectularius* (Heteroptera, Cimicidae)

**DOI:** 10.3897/CompCytogen.v10i4.10681

**Published:** 2016-12-14

**Authors:** David Sadílek, Robert B. Angus, František Šťáhlavský, Jitka Vilímová

**Affiliations:** 1 Charles University in Prague, Faculty of Science, Department of Zoology, Viničná 7, CZ-12844 Praha, Czech Republic; 2 Department of Life Sciences (Entomology), The Natural History Museum, Cromwell Road, London SW7 5BD, UK

**Keywords:** holokinetic chromosomes, spreading method, squashing method, testes, midgut, karyogram

## Abstract

In the article we summarize the most common recent cytogenetic methods used in analysis of karyotypes in Heteroptera. We seek to show the pros and cons of the spreading method compared with the traditional squashing method. We discuss the suitability of gonad, midgut and embryo tissue in *Cimex lectularius* Linnaeus, 1758 chromosome research and production of figures of whole mitosis and meiosis, using the spreading method.

The hotplate spreading technique has many advantages in comparison with the squashing technique. Chromosomal slides prepared from the testes tissue gave the best results, tissues of eggs and midgut epithelium are not suitable. Metaphase II is the only division phase in which sex chromosomes can be clearly distinguished. Chromosome number determination is easy during metaphase I and metaphase II. Spreading of gonad tissue is a suitable method for the cytogenetic analysis of holokinetic chromosomes of *Cimex lectularius*.

## Introduction

Insect chromosome research is more than 130 years old (White 1973). Large polytene chromosomes of Diptera (*Chironomus* spp., *Drosophila* spp., *Sciara* spp.) were the first subjects studied (e.g. Korschelt 1884, Koller 1935). Gradually, cytogenetic studies became more and more common, so that now insect cytogenetics is a well-established field of science using various modern enhanced methods (e.g. Cabral-de-Mello et al. 2010, Novotná et al. 2011, van’t Hof et al. 2011, Bueno et al. 2013).

Historically, classical histology was the first method used for preparing arthropod chromosomes, including insect ones, when the tissue in paraffin wax was cut into sections 7-20 microns in thickness (McClung 1899, Montgomery 1901, Darlington 1939, Slack 1939, Parshad 1957, Piza 1957, and others). This method is no longer used for study of insect chromosomes. The next method developed was a squashing technique (Sáez 1950), which began to be widely used in second half of the 20^th^ century (e.g. Leston 1957, Piza 1957, Warren et al. 1960, Ueshima 1963) and it is still considered as a classical method by the majority of insect cytogeneticists including heteropterologists (e.g. Bressa et al. 2002a, 2003, Poggio et al. 2006, Grozeva et al. 2010, Yang et al. 2012).

The most recent method “hotplate spreading” (only spreading hereinafter) was originally used only for vertebrate chromosomes studies. The whole method was then modified by Crozier (1968) and used also for insect chromosome research (namely ants and dipterans). Traut (1976) developed other modifications of the spreading for lepidopteran chromosome analysis. Recently, this method has been used more frequently not only for study of chromosomes of various insect taxa (Bressa et al. 2009, 2015, van’t Hof et al. 2011, Paladino et al. 2013, Sadílek et al. 2013, Chirino et al. 2015) but also for all other main arthropod evolutionary lineages such as arachnids (e.g. Šťáhlavský and Král 2004, Forman et al. 2013, Adilardi et al. 2014, Sadílek et al. 2015), myriapods (e.g. Green et al. 2016), and crustaceans (e.g. Kořínková and Goldyn 2011).

However, the use of the squashing method still strongly prevails over spreading in present Heteroptera cytogenetic studies. As the first, Angus (1982) optimised spreading after Crozier (1968) and applied it for chromosome analysis of Hydrophilidae (Coleoptera). Angus routinely used colchicine to block spindle formation. Later, this method was used in the study of the nepomorphan families Notonectidae (Angus et al. 2004) and Corixidae (Waller and Angus 2005). The spreading technique was also modified by Traut (1976) for male and female meiotic studies in lepidopteran species. Following his procedure, spreading was used successfully for chromosome studies of other heteropteran taxa, namely Corixidae: Micronectinae (Ituarte and Papeschi 2004), Reduviidae: Hammacerinae (Poggio et al. 2011), Triatominae (Morielle-Souza and Azeredo-Oliveira 2007, Poggio et al. 2013a), Reduviinae (Poggio et al. 2013b), Coreidae (Bressa et al. 2008), Pyrrhocoridae (Bressa et al. 2009), and Belostomatidae (Bardella et al. 2012, Chirino et al. 2013, 2014).

One of the very frequently studied Heteroptera is the obligatorily ectoparasitic genus *Cimex* Linnaeus, 1758 (Cimicidae), which includes parasitologically and medically important species. This genus is characterised by possession of the all-important heteropteran cytogenetic features: holokinetic chromosomes (e.g. Wolf et al. 1997, Mola and Papeschi 2006, Papeschi and Bressa 2006, Guerra et al. 2010, Poggio et al. 2014), achiasmatic male meiosis of collochore type (Nokkala and Nokkala 1983, Nokkala and Grozeva 2000, Grozeva and Nokkala 2002, Ituarte and Papeschi 2004, Grozeva et al. 2008, 2010, Poggio et al. 2009, 2014, Kuznetsova et al. 2011), postreductional inverted male sex chromosome meiosis (Viera et al. 2009, Kuznetsova et al. 2011), and the diffuse stage (Kuznetsova and Maryańska-Nadahowska 2000, Bressa et al. 2002b, Rebagliati et al. 2005, Lanzone and Souza 2006). However the cytogenetic research on *Cimex* species is difficult because of some other chromosome characteristics, such as the small size, high morphological similarity and superspiralization during almost the whole period of chromosomal division (e.g. Ueshima 1966, Manna 1984). Holokinetic chromosomes lack a primary constriction and thus a localized centromere, which facilitates structural rearrangements of the karyotype by non-lethal chromosomal fusions and fragmentations. Fusions in this type of chromosomes do not result in dicentric chromosomes. Chromosome fragments are able to attach to spindle fibres and migrate normally during mitosis or meiosis, which enables them to go through further cell division (e.g. Motzko and Ruthmann 1984, Howe et al. 2001, Mandrioli and Manicardi 2003, Schvarzstein et al. 2010).

In addition to the above mentioned features, the important human ectoparasite model species *Cimex lectularius* Linnaeus, 1758 shows intraspecific variability in number of sex chromosomes from three (X_1_X_2_Y) to 21 (X_1_X_2_Y+18 extra Xs) (e.g. Darlington 1939, Slack 1939, Ueshima 1966, Sadílek et al. 2013). In the family Cimicidae, the sex is determined by the presence of an XX/XY (female/male) simple sex chromosome system in 53 cytogenetically analysed species. Most cimicid species, including the majority of *Cimex* species, also possess a multiple sex chromosome system X_1_X_1_X_2_X_2_/X_1_X_2_Y (except *Cimex antennatus* Usinger & Ueshima, 1965, *Cimex latipennis* Usinger & Ueshima, 1965 and *Cimex incrassatus* Usinger & Ueshima, 1965 with the basic XX/XY system) (Poggio et al. 2009, Grozeva et al. 2010, Kuznetsova et al. 2011, Sadílek et al. 2013). Four cimicid species possess constantly three X chromosomes (X_1_X_2_X_3_Y, male) (*Paracimex capitatus* Usinger, 1966, *Paracimex inflatus* Ueshima, 1968, *Paracimex philippinensis* Usinger, 1959 and *Hesperocimex coloradensis* List, 1925) and two species four X chromosomes (X_1_X_2_X_3_X_4_Y, male) (*Cimex adjunctus* Barber, 1939 and *Cimex brevis* Usinger & Ueshima, 1965) (Ueshima 1979, Kuznetsova et al. 2011).

Intraspecific variability in the number of X chromosomes has been described in three cimicid species from the subfamily Cimicinae, *Paracimex borneensis* Usinger, 1959 (2X; 5-9X), *Paracimex capitatus* (2-6X) and *Cimex lectularius* (2-20X) (summary in Ueshima 1966, 1968, 1979). The numbers of *Cimex lectularius* X chromosomes can differ among different populations (localities), or among specimens within one population. Even a single specimen can contain cells with different numbers of X chromosomes (Ueshima 1966, 1979, Sadílek et al. 2013). Preliminary study has also indirectly indicated the possibility of a variable number of X chromosomes in an obligatory bat parasite *Cimex pipistrelli* Jenys, 1839 (Sadílek et al. 2013). Therefore, it seems possible that intraspecific variability of X chromosomes could be a general feature of the genera *Cimex* and *Paracimex* Kiritshenko, 1913, or even possibly a wider spectrum of Cimicidae species.

*Cimex lectularius* became an intensively studied species by a wide spectrum of scientific approaches due to its recent massive global expansion (e.g. Hwang et al. 2005, Romero et al. 2007, Reinhardt et al. 2008, Weeks et al. 2010, Balvín et al. 2012, Booth et al. 2015), including cytogenetic studies using modern methods by Grozeva et al. (2010, 2011) and the detailed analysis of variable karyotype by Sadílek et al. (2013). As it is generally very important to improve methodological approach to research, we used the spreading method for preparing *Cimex lectularius* chromosome slides.

The main aim of the present study is to compare results of the spreading method, used for the first time in the Cimicidae, with the traditional squashing method. We aimed to find out if the spreading method resulted in different or more conclusive data and could be therefore more suitable for analysis of cimicid holokinetic chromosomes. The use of spreading is currently quite rare even within researches of other Heteroptera species but it is also recommended for cytogenetic studies of the other insect orders. The present paper also makes comparisons of the suitability of different tissues for cytogenetic study, and of distinct cell division phases, chromosome size measurement and assembly of *Cimex lectularius* karyograms.

## Material studied and equipment used

### Material studied

220 specimens of *Cimex lectularius* collected from 65 localities in 10 European countries in the period 2010–2012 were studied, for geographical origins see Sadílek et al. (2013). Live specimens were mostly collected by pest exterminators from human dwellings. They were either studied immediately or were kept alive in the refrigerator at 4 °C without any blood meal. They could survive in good health in such conditions even more than a half of year. Gonad tissue from 115 adult males, 81 adult females and 24 larvae was studied cytogenetically. From those specimens 116 slides of mesenteron (1 slide per specimen) and 13 slides of eggs/embryos (1 slide from a few eggs per female) were also analysed.

### Equipment used

The chromosome slides were examined using the Olympus Provis AX 70 light microscope and selected cells and stages of division were documented by the digital imaging system Olympus DP 72 and software QuickPHOTO CAMERA 2.3. Karyograms were made in graphic editor Corel DRAW X5. For assembly of karyograms, chromosomes were cut out from photographs, measured and sorted by size in software ImageJ 1.47 with Levan plugin (http://imagej.nih.gov/ij/).

## Results and discussion

### Hotplate spreading

The basic principle of the hotplate spreading technique is to turn extracted tissue into a suspension and let cells to adhere to the surface of a microscope slide (optimal is SuperFrost quality slide) as the drop was moved on the slide by pushing it with fine tungsten needles and evaporated. The resulting semipermanent slide (without cover slip) is characterized by its long durability (for years), stored at 4 °C for basic Giemsa staining or -20 °C to -80 °C for further molecular analysis (e.g. FISH). *Cimex lectularius* specimens were dissected in hypotonic solution 0.075 M KCl immediately after killing, to keep the gonad tissue hydrated and remove debris of other tissues. During hypotonisation, the cells receive additional water due to osmosis, making them larger, the contents of the cell are loosened and chromosomes become more individualized. Chromosomes can be damaged or washed away during final dissociation in case of excessive hypotonic treatment. However, chromosomes are still too compact and are not analysable in insufficiently hypotonised cells. Several time periods of tissue hypotonisation were tried: 10, 15, 20, 25 and 30 minutes. The best results were obtained from samples after 25 minutes of fresh hypotonic solution treatment.

Tissue fixation in methanol: glacial acetic acid 3:1 was the next step, methanol can be replaced by 99.9% ethanol. Alcohol causes immediate death of cells and acetic acid penetrates the membrane for quick ideal preservation of inner structures especially chromosomes. Two types of fixation were tested, one step fixation for 5, 10, 15 or 20 minutes, and two step fixation for 5+10, or 10+20 minutes. However, the duration of fixation had a minor effect on the final quality of chromosomes on slides. Two step fixation for 5+10 minutes was found to be optimal, the tissue dehydration effectiveness increased because in the second fixation step dilution by water from hypotonic solution was reduced to minimum.

Fixed tissue was mechanically suspended on the slide with tungsten needles and cells were chemically released by adding of 1–2 drops of 60% acetic acid. Undissociated clusters of tissue were removed. The slides with suspension were put on a warm (45 °C) histological plate and the drop was moved all around the slide with the needle. Adhering cells can create hardly diagnosable clusters without that movement. The chromosome sets are very often overlapping in those clusters. Suspension movement also contributes to evenly distributed chromosomal material on the slide surface. The slides were stained on the second day, allowing them to dry properly and to avoid loss of chromosomes. The staining was carried out using a 5% Giemsa solution in Sörensen phosphate buffer (pH = 6.8) for 10, 15, 20, 30 or 40 minutes (optimum in 30 minutes). The stained slides were stored in a refrigerator at 4 °C. The mechanism of cell adherence is described in detail by Imai et al. (1988).

The squashing technique is the more widely used method in Heteroptera cytogenetics. Usually, living specimens are directly fixed in ethanol: glacial acetic acid or methanol: glacial acetic acid (3:1) and can be stored at 4 °C for later use. Dissected gonad tissue is squashed under a cover slip in a drop of 45% acetic acid, which is then frozen using dry ice (solid CO_2_) (e.g. Kuznetsova and Nadachowska 2000, Grozeva et al. 2010, Kuznetsova et al. 2015), or freezing in liquid nitrogen (e.g. Pérez et al. 2004, Bardella et al. 2010) to allow removal of the coverslip. There are also frequent modifications of squashing method for example with use of acetic haematoxylin (e.g. Bressa et al. 2005) or iron propionic haematoxylin (e.g. Rebagliati et al. 2001). After removing cover slips with a razor blade, the slides are dehydrated in fresh fixative for 15 min and air-dried (e.g. Grozeva and Nokkala 2002, Grozeva et al. 2010). The slides are stained with Feulgen Giemsa (e.g. Grozeva and Nokkala 1996).

An undoubted advantage of the squashing method is a possibility to fix material right in the field and then keep it in 70% ethanol at 4 °C for a long time (months, years), but the gonads kept longer period in cold become harder and the squashing of tissue would be more difficult. Material cannot be preserved before use of spreading method, because chromosomes from fixed cells cannot be spread. The major advantage of spreading is easier methodology. In particular, independence of dry ice or liquid nitrogen (hard to supply in the field) makes it possible to use this method outside the laboratory, with the only demand being for electricity or even without hotplate at the room temperature - higher temperature fasten the evaporation and the efficiency of the plate spreading technique.

The spreading needs manual skill in suspension droplet movement on slide after dissociation. Unsuitable manipulation could lead to loss, damage or overlap of chromosomes. On the other hand, a squashed tissue could be easily insufficiently spread and then the chromosomes on slides could be poorly, or not at all analyzable, or even the tissue can be lost during coverslip removing. The use of squashing can be very problematic in organisms with high chromosome number.

The spreading is generally an easier technique, which provides slightly better results than the squashing technique and often provides abundant slides with well-dispersed cells suitable for further analysis. Therefore, gonad tissue spreading is a suitable method for the cytogenetic analysis of Heteroptera, particularly with focus on the small, variable and numerous holokinetic chromosomes of *Cimex lectularius*. The main advantages and disadvantages of the two methods are summarised in Table 1.

**Table 1.** Summary of general advantages and disadvantages of the hotplate spreading and squashing methods of chromosome preparation.

	Spreading	Squashing
**Material**	- must be killed freshly	+ can be fixed in field
	- keep it alive, store it for short time (month)	+ store it for months or longer
**Equipment**	+ possible to perform it in the field (need of electricity)	- not possible to perform it in the field (need of solid CO_2_ or liquid N)
**Overall difficulty**	+ lower	- higher
	+ just handle to move with droplet on slide properly with fine tungsten needles	- cells must be in chromosomes on slide is hardly analyzable single layer, if not
**Results**	+ even on material rich slides is only single layer of cells	- on material rich slides is high probability of overlap
	+ i.e. more analyzable nuclei	- i.e. fewer analyzable nuclei

### Tissue suitability and results obtained

*Cimex lectularius* reproduction is acyclic, which is why it is almost impossible to find out the exact age or physiological condition of wild specimens. Negative results from specimens with inactive gonads (absence of cell division) could be caused just by starving. Exact age and condition could be known only in laboratory reared specimens and it is also possible to use eggs or larvae of specific age.

Chromosome slides were made from tissues with the highest mitotic index, which express amount of dividing cells. Meiotic chromosomes could be isolated only from gonad tissue, but mitotic chromosomes should be obtained from all types of proliferating tissues as in insect e.g. hemolymph, epithelium of digestive tract and in holometabolous insect imaginal disc.

**Figure 1.**
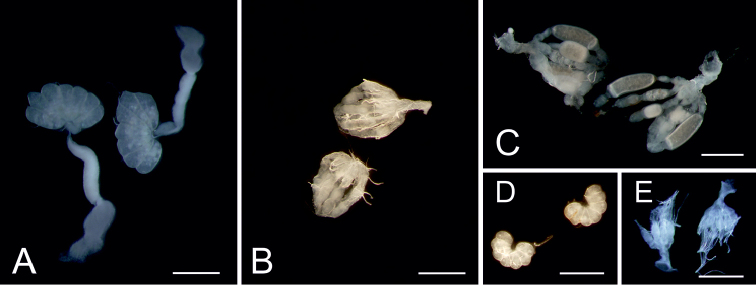
Adult and 5^th^ instar larva *Cimex lectularius* gonads. **A** Adult testes **B** Adult ovaries, without eggs **C** Adult ovaries, with well-developed eggs **D** 5^th^ instar larva testes, well-developed, probably sub adult specimen **E** 5^th^ instar larva ovaries. Scale bar = 1 mm.

**Figure 2.**
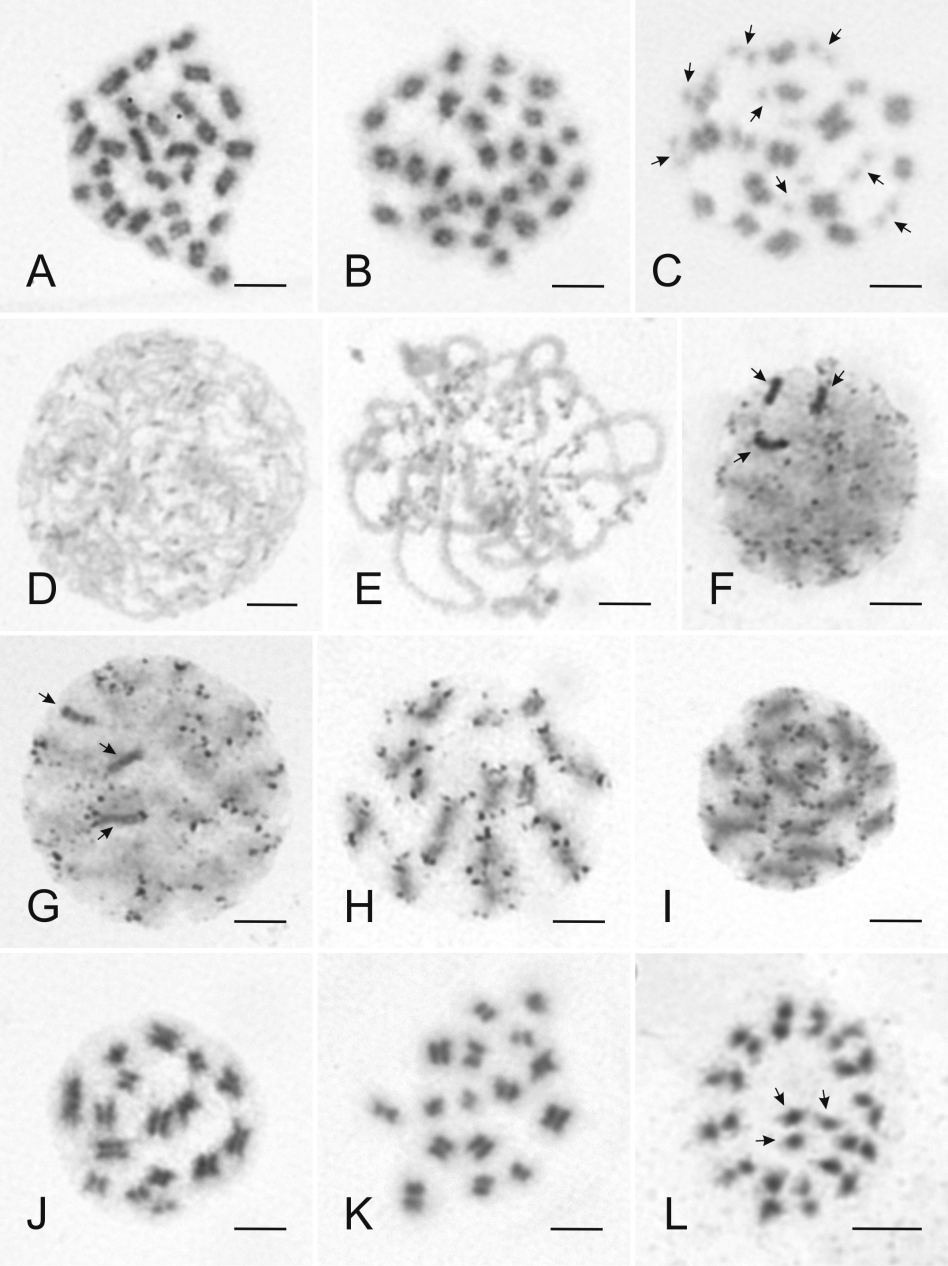
Various stages of mitotic and meiotic *Cimex lectularius* chromosomes with basic karyotype 2n = 26+X_1_X_2_Y (**A, B, D–L**) and karyotype 2n = 26+X_1-10_Y (**C**), stained with Giemsa. **A** Mitotic prometaphase ♂ **B** Mitotic metaphase ♂ **C** Metaphase I ♂ **D** Leptotene ♀ **E** Pachytene ♀ **F** Diffuse stage ♂ **G** Diffuse stage - postpachytene transition ♂ **H** Postpachytene ♂ **I** Late postpachytene ♂ **J** Prometaphase I ♂ **K** Metaphase I ♂ **L** Metaphase II ♂. Arrow = sex chromosome (**F, G, L**) or fragments of supposedly sex chromosomes (**C**). Scale bar = 5 μm.

**Gonads.** Generally, tissue of gonads is used for cytogenetical studies, mainly testes (Fig. 1A, D) (e.g. Kuznetsova et al. 2004, Bressa et al. 2009, Grozeva et al. 2010, Poggio et al. 2011, 2014), sometimes ovaries (Fig. 1B, C, E) (e.g. Angus et al. 2004, Waller and Angus 2005). We obtained chromosomes in all various stages of spermatogenesis (mitosis and meiosis) from *Cimex lectularius* testes, and only mitotic chromosomes in its ovaries. However, also frequent meiotic pachytene cells (Fig. 2E) were recorded in ovaries. This could mean that the female pachytene is a prolonged resting phase when immature oocytes stop meiosis until feeding or mating. In the contrast to females, the pachytene stage in *Cimex lectularius* males is very short and its finding is extremely rare.

Gonads from 4^th^ and 5^th^ instar larvae were analyzed as well as those of adults (Fig. 1A–C). Gonads of the 4^th^ larval instar are always very small, any manipulation of them is quite difficult as well as a correct determination of sex. Size of the 5^th^ larval instar gonads (Fig. 1D, E) can be different in wide spectrum, from miniature as in the 4^th^ larval instar to large and well developed in sub adult specimens, in which also sex can be distinguished easily. In the older 5^th^ instar larvae, nuclei from mitosis to meiotic metaphase II can be seen (Fig. 2A, B, L).

In *Cimex lectularius* feeding directly initiates mating behaviour and cell division in gonads, thus this is a required condition for gonad growth and gamete production (Usinger 1966). In our study, small gonads and therefore negative slides were recorded even from recently (approximately 7 days) fully engorged specimens, which probably could not digest their meal and start gamete production.

Testes tissues were shown to be very suitable for the *Cimex lectularius* cytogenetic research. They contain large quantities of cells in all stages of meiotic and mitotic division and provide enough information for complete karyotype analysis. Ovarian tissue is suitable in cases of lack of males or as a reference in samples with a higher chromosomal variability, and to confirm the sex chromosome system in comparison with chromosomes of males. In samples of *Cimex lectularius* with variable karyotype, it is interesting to observe complementarity of chromosome number between males and females, and it is also possible to study females with varying X chromosome numbers in oocytes, originating from breeding of specimens with different karyotypes (Sadílek et al. 2013).

The absence of meiotic metaphases in adult females suggests meiotic division in an earlier instar. However, because of quite frequently recorded pachytene nuclei (Fig. 2E) the whole meiotic division has to take place even in adult females. In the contrast, in testes pachytene chromosomes were recorded very rarely, thus it is possible to propose a different length of the pachytene stage between sexes. It is very possible that the whole phenomenon is connected to male achiasmatic meiosis. Male pachytene checkpoint is missing because of male recombination absence that means the male pachytene is very fast and hard to record (e.g. Tung et al. 2000).

Heteroptera cytogenetics is studied usually on male gonads. Detailed study of female karyotype is often problematic, because there is much lower abundance of dividing cells in ovaries than in testes and because all female meiotic stages are almost impossible to record. These are the main reasons for the absence of information about female cytogenetics especially meiosis (Kuznetsova et al. 2011). Nevertheless, in a case of complicated variable karyotypes of *Cimex lectularius*, the analysis of female cytogenetics results is important and highly recommended.

**Midgut epithelium.** This tissue should be suitable for cytogenetic study due to continual wasting of digestive cells, followed by intensive mitotic division and differentiation of the regenerative (= stem) cells (e.g. Azevedo et al. 2009, Rost-Roszkowska et al. 2010a, 2010b). Nevertheless, midgut epithelium slides of *Cimex lectularius* contained no countable mitotic chromosomes. Even specimens with rich chromosome slides from gonads provided no records of any chromosomal division in midgut epithelium. We found only one poor nucleus with mitotic chromosomes from 116 slides analysed. It is very interesting that absence of mitosis in midgut is not connected either with presence or absence of food in midgut lumen. Negative slides without distinct particular chromosomes for karyotype study were from specimens with completely full, through all situations, to empty midgut. It could be similar to the case recently described in Ceratopogonidae (Diptera) (Urbanek and Rost-Roszkowska 2015). In studied dipterans, gonad maturing induces degeneration of digestive cells of midgut epithelium, which are used as accumulated nutrients and not apparently replaced, because in adult females regenerative cells are very rare. The mitotic division of regenerative cells has not been observed even in larvae in this case, in which the cells are only differentiated. Recently, no mitotic divisions and differentiations of the regenerative cells were observed in midgut epithelium of two *Cimex* species, *Cimex lectularius* and *Cimex pipistrelli* (Rost-Roszkowska et al. 2016).

It is more complicated to obtain mitotic chromosomes from midgut epithelium than from gonad tissue in general. The use of colchicine or other mitosis-inhibiting agents, which abolish spindle formation and leave the chromosomes free in the cell, as in the studies of Angus et al. (2004) and Waller and Angus (2005) is necessary for clear chromosome preparations. Colchicine is not a mitostatic when applied to whole insects or embryos, but allows the chromosomes to continue their mitotic cycle (contraction, separation of chromatids, re-elongation) while lying free in the cell. However, our completely negative results suggest that the *Cimex lectularius* midgut tissue is not suitable for chromosome research even with colchicine treatment.

**Eggs.** This stage of insect generally contains many tissues with a large amount of mitotic cells of the growing embryo. However, we were not successful in recognizing of these cells on spreaded slides. Three low quality mitoses were recorded on only a single slide from 13 slides analyzed. A serious complication is the unpredictable presence of eggs in wild *Cimex lectularius* females, and also the impossibility of distinguishing in advance sex of the embryos. We are sure the sex of embryos only in case of the male basic karyotype 2n = 26+X_1_X_2_Y, otherwise we are not able to distinguish between male with one more supernumerary chromosome (X_1_X_2_X_3_Y) and basic karyotype of female 2n = 26+X_1_X_1_X_2_X_2_.

The use of eggs is not common in Heteroptera cytogenetics, but for example in study of holokinetic chromosomes in parthenogenetic Psocoptera (Nokkala and Golub 2006), parthenogenetic psyllids and of monocentric chromosomes of Hydrophilidae (Coleoptera) was use of embryonic tissues successful (Angus 1982, Shaarawi and Angus 1991). However, the authors in Hydrophilidae studies used a different modified spreading technique after Crozier (1968).

The karyotype was successfully determined in 128 out of 220 specimens of *Cimex lectularius* (58%), 80 males and 48 females, from 140 positive chromosomal slides (34%) (with cells in division) out of 412 examined. Slides prepared from testes tissue gave the best results, 90 positive slides out of 170 (53%). Ovarian tissue contains only mitosis with a lower number of 50 positive slides out of 111 (45%). However, the tissues of midgut and eggs were surprisingly unsuccessful, with only 2 positive slides out of 125 (1.6%). All slides were treated identically, therefore a ratio between positive and negative slides could show percentage of specimens in ideal physiological state for getting mitotic and meiotic chromosomes.

### Chromosome division phases studied

The following stages of cell division were observed with various frequencies in *Cimex lectularius* males. Mitotic cells were recorded especially in metaphase and prometaphase stages (Fig. 2A, B) in 80% of specimens. Leptotene and pachytene stages were detected only in two specimens. In late prophase I, the most frequent meiotic cells were diffuse stage in 90% of specimens and postpachytene in 30% of specimens (Fig. 2F–I). Less frequently, cells in metaphase I (Fig. 2K) were observed in 25% of specimens, and cells in metaphase II (Fig. 2L) were the most rare, only in 20% of specimens. Metaphases I were frequently very abundant in the specimens, in a contrast short lasting stages of prometaphase I (Fig. 2J) and II were observed always in small amounts and only in a four specimens.

On slides from ovary cells in mitotic metaphase stage (100% of specimens) only early prophase I (leptotene and pachytene) (Figs 2D, E) from meiotic division were detected. Leptotene nuclei were recorded only in 10% of specimens, pachytene nuclei were observed in 50% of specimens in small densities only. In females no cells were observed in late meiosis, which was the main stage of male cells.

Leptotene (Fig. 2D) and pachytene (Fig. 2E) nuclei are isopycnotic and did not show any distinct features. At diffuse stage (Fig. 2F), autosomes are decondensed and the sex chromosomes are distinctly positive heteropycnotic. During transition from diffuse stage to postpachytene (Fig. 2G), the sex chromosomes become isopycnotic and cannot be distinguished from autosomes. Postpachytene may be considered as meiotic prophase stage that substitutes diplotene and diakinesis in organisms with achiasmatic meiosis where no recombination occurs. During postpachytene, autosomes condensate again and dark terminal spots on telomeric regions of each chromatid appear (Fig, 2G–I). The dark spots disappear at the end of postpachytene, and from prometaphase onwards the chromosomes are isopycnotic (Fig. 2J) and continue in condensation until metaphase I.

In metaphase I (Fig. 2K), nucleus with basic karyotype 2n = 26+X_1_X_2_Y shows 13 autosomal bivalents and three sex chromosomes, which do not pair with each other. Male metaphase II is radial, the 13 autosomes dispose in a ring configuration and the X_1_, X_2_ and Y chromatids form a pseudotrivalent, which lies at the centre of it (Fig. 2L), in concordance with observation of Ueshima (1967), Grozeva et al. (2010) and Sadílek et al. (2013). Metaphase II is the only stage in which it is possible to definitely distinguish autosomes and sex chromosomes. The chromosome arrangement of metaphase II precisely matches the inverted meiosis of sex chromosomes, in which reductional division of autosomal bivalents occurs in anaphase I whereas the sex chromosomes segregate chromatids (equational division). In anaphase II, autosomes segregate sister chromatids, and the X_1_ and X_2_ chromatids segregate from the Y (Ueshima 1966, 1979, Grozeva et al. 2010), even with 20 X supernumerary sex chromosomes (Sadílek et al. 2013) (Fig. 2C). Only the metaphase II reflects clearly number of sex chromosomes in *Cimex lectularius* with supernumerary sex chromosome fragments, because this is the only phase where autosomes and sex chromosomes can be distinguished.

Chromosome number determination is notably easier in meiotic metaphase I and II than in mitosis, because chromosomes are paired and superspiralized. These results show that, using the spreading method, it is possible to get mitotic and meiotic chromosome slides in high quality for further analysis.

### Karyogram assembly

Well-spread mitotic chromosomes can be used to assemble karyograms. A particular requirement here is that chromosomes are not physically stretched in the course of preparations, as can happen with squashes. A karyogram represents standard format of species karyotype image that helps us to distinguish chromosomes, generally specific pairs of autosomes and sex chromosomes (e.g. Angus et al. 2015). The chromosomes are usually ordered by size, position of centromere and some specific marker on chromosomes (e.g. C-bands, G-bands, DAPI/CMA_3_ fluorescent bands, Ag-NOR bands or position of specific genes visualized by FISH) (Marco et al. 2009, Maryańska-Nadachowska et al. 2012, Chirino et al. 2015). However, in Heteroptera the holokinetic organization and the chromosome composition do not allow to use many of these cytogenetic features. Besides, some characters may be so variable that the comparison is very complicated, especially between different stages of mitotic or meiotic divisions. For example, the size of chromosomes can vary distinctively according spiralization in various phases. That is a reason the relative size of chromosomes (percentage of single chromosome length from whole karyotype) is used more frequently. In this case, it is necessary to measure all the chromosomes in a great number of cells at the same division stage, i.e. at metaphase I, or at metaphase II, or at spermatogonial metaphase (e.g. Sakamoto and Zacaro 2009, Chirino et al. 2013, 2014, Sadílek et al. 2015).

**Figure 3.**
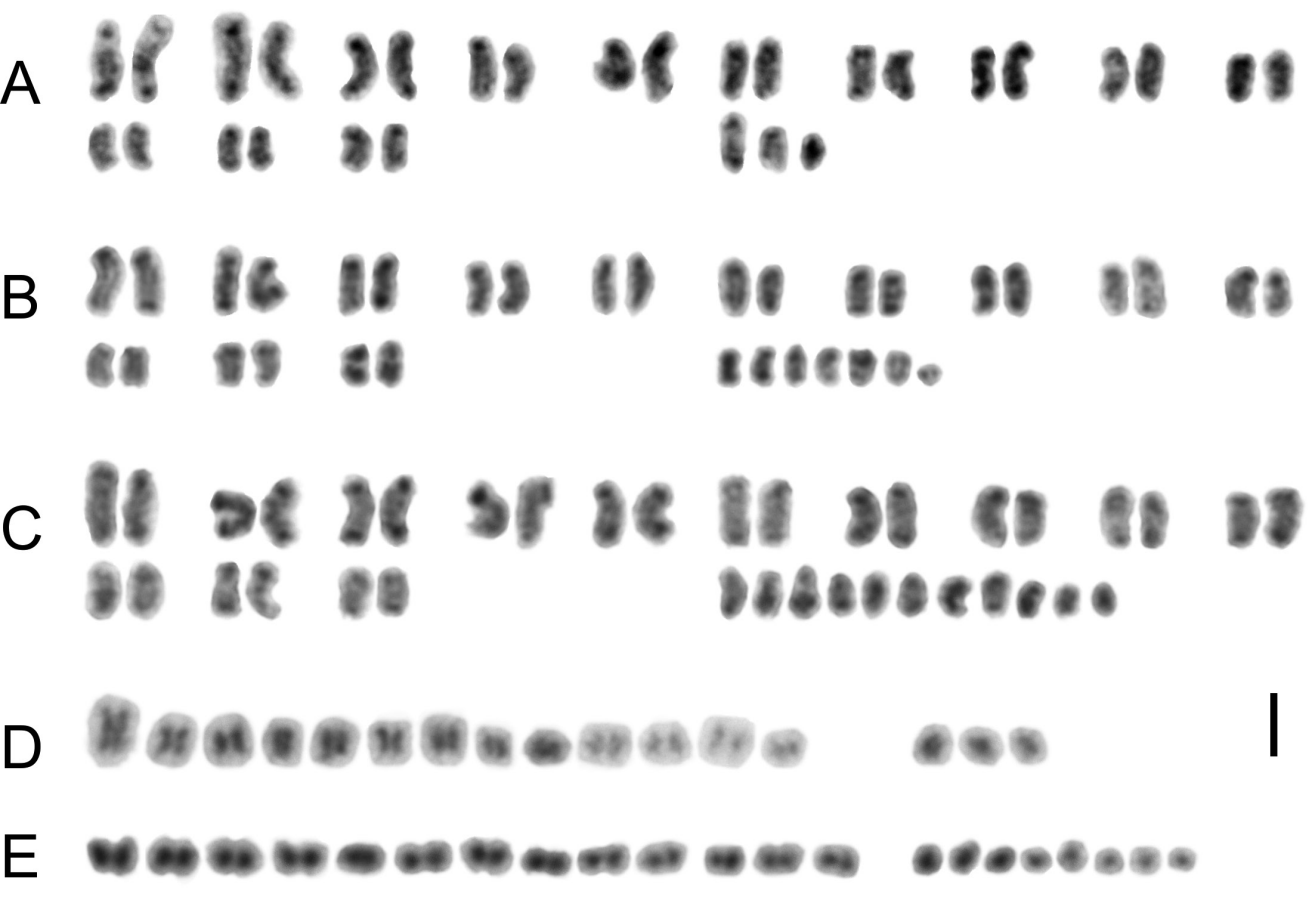
Male mitotic and meiotic karyograms of *Cimex lectularius* chromosome variants. **A-C** Mitotic prometaphase. **A** 2n = 26+X_1_X_2_Y **B** 2n = 26+X_1-6_Y **C** 2n = 26+X_1-10_Y **D** Prometaphase II, 2n = 26+X_1_X_2_Y **E** Metaphase II, 2n = 26+X_1-7_Y. Scale bar = 5 µm.

We assembled three examples of karyograms from *Cimex lectularius* mitotic chromosomes from different chromosome number of 2n = 29, 33 and 37 (Fig. 3A–C) and two male meiotic karyograms from prometaphase II and metaphase II, 2n = 26+X_1_X_2_Y (Fig. 3D) and 2n = 26+X_1-7_Y (Fig. 3E), respectively. In these cases the size of chromosomes was measured trying to find out the fragmentation events - decreasing size of the X chromosomes during increasing of their number. Nuclei in mitotic prometaphase provide the most relaxed and still quite compact chromosomes, thus the best stage for getting karyograms (Fig. 3A–C). In the contrast, chromosomes in both meiotic metaphases I and II are globular and very similar to each other (Fig. 3D, E). Moreover, *Cimex lectularius* chromosomes do not show any strong morphological pattern, and distinguishing pairs of autosomes and sex chromosomes is not easy. In the mitotic prometaphase, we are able to put together some chromosomal pairs according to heteropycnotic regions on the ends of chromosomes visible just after regular Giemsa stain (Fig. 3A–C). The pattern of chromosomes change a little among different karyograms, so it is not possible to use it as a clear diagnostic feature. The sex chromosomes can be quickly recognised only in metaphase II (Figs 2L, 3D, E).

In each of *Cimex lectularius* karyotypes the size of chromosomes gradually decreases. That is a reason why the size expressed only as a percentage is not very suitable for karyotype comparison among congeneric species with different diploid chromosome numbers because they have different distribution of length. However, in case of *Cimex lectularius* chromosome fragments we can predict their very small size as on example of metaphase I with 2n = 26+X_1-10_Y (Fig. 2C). In the contrast two karyograms show additional sex chromosomes (Fig. 3B, C) of almost the same size as the sex chromosomes in karyotype with basic chromosome number (Fig. 3A). It suggests an occurrence of non-disjunction or even possibility of chromosome fragments different spiralization. Another explanation could be the fragments resulted from fragmentation in different parts of the original sex chromosomes. If fragments origin is independent in various populations, they simply cannot be identical and must differ by size and content. All these hypotheses need further study. The karyogram assembly brought us at least rough chromosome size comparison of *Cimex lectularius* various karyotypes.

## Conclusion

The hotplate spreading technique has many advantages in comparison with the squashing technique. It is suitable for use by cytogenetic beginners as they need only to get the manual skill in suspension droplet movement on slide. One disadvantage of spreading exists – material has to be prepared freshly after killing, either in the field or after keeping alive in a lab. However, *Cimex lectularius* is capable to survive in good health several months without any meal. The spreading technique seems to be ideal for study of specimens with numerous holokinetic chromosomes.

Tissue of testes, the usual material for insect cytogenetic studies, appeared to be the most suitable also in chromosome study of *Cimex lectularius*. Ovaries sometimes also show some interesting results. But the tissue of midgut and eggs – supposedly suitable, did not show any satisfactory results.

Results based on ovarian tissue could be easily misinterpreted. During dissociation, cells from ovaries and developing male and female embryos resulted from mating with unknown karyotype male could be mixed. Thus it is possible to observe artificial heterogenic sample of three karyotypes, which can be misleadingly considered as a variability in one female karyotype. This is made possible thanks to cimicid specific traumatic insemination and egg fertilization directly in ovarioles, whole effect could be also magnified by low abundance of mitotic nuclei in ovarian tissue in general. To avoid this problem would be necessary to separate only germarium, part of ovaries where mitosis give rise to primary oocytes.

Meiotic metaphase II is the best division phase for study of chromosomes in *Cimex lectularius*, but mitotic prometaphase and metaphase I are also usable. Our suggestion that the abundant nuclei in diffuse stage could serve for quick diagnosis of sex chromosome number was not proved. Nuclei of specimens with higher number of sex chromosomes did not show clear number of heteropycnotic sex chromosome elements. The explanation could be either that spiralization of sex chromosome fragments has changed so they are no more positively heteropycnotic during diffuse stage, or too small size of fragments.
